# The *Edwardsiella* T3SS effector EseQ promotes invasion by altering the cell’s cytoskeleton and disrupting the epithelial barrier

**DOI:** 10.1128/msphere.00520-25

**Published:** 2025-11-18

**Authors:** Xiu Long Jiang, Tian Tian He, Pu Yu Tang, Qin Wang, Pin Nie, Hai Xia Xie

**Affiliations:** 1 State Key Laboratory of Breeding Biotechnology and Sustainable Aquaculture, Institute of Hydrobiology, Chinese Academy of Sciences 53021, Wuhan, China; 2 College of Advanced Agricultural Sciences, University of Chinese Academy of Sciences 74519, Beijing, China; University of Wyoming, Laramie, Wyoming, USA

**Keywords:** T3SS effector, cell cytoskeleton, epithelial barrier, invasion, *Edwardsiella piscicida*

## Abstract

*Edwardsiella piscicida* is a Gram-negative, intracellular, enteric pathogen that primarily causes hemorrhagic septicemia in fish. It survives and replicates within host cells by delivering a subset of effector proteins via the type III secretion system (T3SS). Previous research has identified a novel T3SS effector, EseQ, in *E. piscicida;* however, its function remains unclear. This study reveals that EseQ binds to both α-tubulin and GEF-H1 (Rho guanine nucleotide exchange factor 1), causing microtubule destabilization and the release of activated GEF-H1. Active GEF-H1 then stimulates the conversion of GDP-RhoA (the inactive form) to GTP-RhoA (the active form), which subsequently induces stress fiber formation in a ROCK-dependent manner. Stress fibers induced by EseQ alter the architecture of zonula occludens-1-mediated intercellular junctions. This leads to increased permeability of the epithelial barrier, thereby facilitating the translocation of *E. piscicida* through epithelial cell layers and its invasion into zebrafish larvae. In conclusion, this study demonstrates that the *Edwardsiella* T3SS effector protein EseQ promotes invasion by manipulating the microtubule and actin cytoskeletons and by disrupting the epithelial barrier.

**IMPORTANCE:**

*Edwardsiella piscicida* causes severe hemorrhagic septicemia in marine and freshwater fish worldwide, resulting in significant economic losses for the aquaculture industry (K. Y. Leung, Q. Wang, Z. Yang, and B. A. Siame, Virulence 10:555–567, 2019, https://doi.org/10.1080/21505594.2019.1621648). Our previous research identified a novel type III secretion system effector, EseQ, in *E. piscicida* whose function remains to be elucidated. In this work, we showed that EseQ binds to tubulin and GEF-H1 and destabilizes microtubules. GEF-H1 released from microtubules activates the RhoA-ROCK-MLCII signaling pathway, leading to stress fiber formation in epithelial cells. EseQ deforms the epithelial barrier and promotes *E. piscicida*’s invasion in a stress fiber-dependent manner. This work contributes to the understanding of the mechanism by which *E. piscicida* invades host cells.

## INTRODUCTION

*Edwardsiella piscicida* PPD130/91 (previously known as *Edwardsiella tarda* PPD130/91) is a Gram-negative intracellular bacterium that causes severe hemorrhagic septicemia primarily in marine and freshwater fish species worldwide (1). It is also emerging as a cause of gastrointestinal and extraintestinal infections in humans (2). *E. piscicida* replicates in epithelial or macrophage cells in a type III secretion system (T3SS)-dependent manner (3, 4).

The T3SS is a complex nanomachine that delivers effector proteins from the bacterial cytosol into host cells (5). T3SS effectors play a role in invasion and phagosomal escape, as well as in the inhibition of innate immune signaling and cell-to-cell spread (6). Identifying and characterizing bacterial virulence proteins helps us understand microbial pathogenicity and aids the development of therapeutic strategies. Recently, 11 new T3SS effectors were identified in *Edwardsiella piscicida* using a method that combines language embedding and biological feature analysis to predict effectors across modalities (7). This is a significant addition to the six characterized T3SS effectors. Among them, EseG binds to α-tubulin and disassembles microtubules (8); EseJ facilitates the replication of *E. piscicida* by inhibiting the production of reactive oxygen species and preventing *E. piscicida* from reaching lysosomes via endocytic trafficking (9, 10); EseK and EseH promote colonization and virulence by targeting the host’s MAPK signaling pathways (11, 12). Trx2 suppresses host apoptosis and inhibits the activation of the NF-κB pathway (13). YfiD, meanwhile, inhibits the activation of host poly(ADP-ribose) polymerase-1 to promote bacterial infection (14).

The EsaB/EsaL/EsaM complex controls the secretion of T3SS effector proteins (15). EsaN is the T3SS ATPase responsible for energizing the secretion of T3SS proteins (8). In our previous work, we identified the novel T3SS effector EseQ in *E. piscicida* by comparing the secretome between the Δ*esaB* strain and the Δ*esaB*Δ*esaN* strain (13). Preliminary sequence analysis using Swiss-Model indicates that EseQ has a tertiary structure homolog to that of the *Shigella* T3SS effector IpgB2, suggesting an association with the host cell cytoskeleton. Most intracellular bacteria invade by manipulating the host cell’s cytoskeleton (16, 17). The *Shigella* T3SS effector IpaA binds to the focal adhesion protein vinculin. This induces cytoskeletal rearrangements that facilitate bacterial invasion (18). The *Salmonella* pathogenicity island 1 (SPI-1) effectors SipA and SipC promote the polymerization of actin, which leads to the internalization of *Salmonella* (19).

The cellular cytoskeleton comprises three types of filaments: actin filaments, microtubules, and intermediate filaments; the functional dynamics and structural organization of microtubules and actin are closely linked (20). The arrangement and stability of microtubules and actin are regulated by the Rho family of small GTPases (21). This family comprises Cdc42, Rac1, and RhoA and is also referred to as Rho GTPases. Activation of RhoA induces cell contraction by promoting the formation of stress fiber bundles and focal adhesions (22, 23), Cdc42 promotes the formation of filopodia, whereas Rac1 facilitates the formation of lamellipodia (22, 23).

Rho GTPases have emerged as a key target for bacterial effectors that manipulate the cell’s cytoskeleton (17, 24, 25). The binding and hydrolysis of GTP cause RhoA to change from an active, GTP-bound state to an inactive, GDP-bound state (26). This transition is mediated by guanine nucleotide exchange factors, such as GEF-H1 (21). The *Bartonella* T4SS effector BepC recruits GEF-H1 to the plasma membrane, thereby inducing the formation of stress fibers through the activation of RhoA (27). The *Vibrio parahaemolyticus* T3SS effector VopO binds to GEF-H1, thereby promoting the formation of stress fibers by activating the RhoA-ROCK signaling pathway (28). Once activated, RhoA binds to its effector, ROCK, thereby propagating downstream signaling. ROCK then regulates the phosphorylation of myosin light chain (MLC), thereby controlling actomyosin contraction and promoting stress fiber formation (26, 29).

Cytochalasin D, a specific inhibitor of actin polymerization, strongly blocks the internalization of *E. piscicida* (30). This suggests that some effectors may facilitate *E. piscicida* invasion by disrupting the host’s actin cytoskeleton. EseQ is a recently identified T3SS effector in *E. piscicida* (13), the function of which remains to be elucidated. This study demonstrates that EseQ binds to tubulin, which destabilizes microtubules and releases GEF-H1. Free, activated GEF-H1 stimulates the formation of high levels of stress fibers in the host cell by activating the RhoA-ROCK-MLCII pathway. EseQ promotes invasion by modulating the microtubule and actin cytoskeletons, thereby causing epithelial barrier dysfunction.

## RESULTS

### Sequence analysis of EseQ

**Fig 1:**
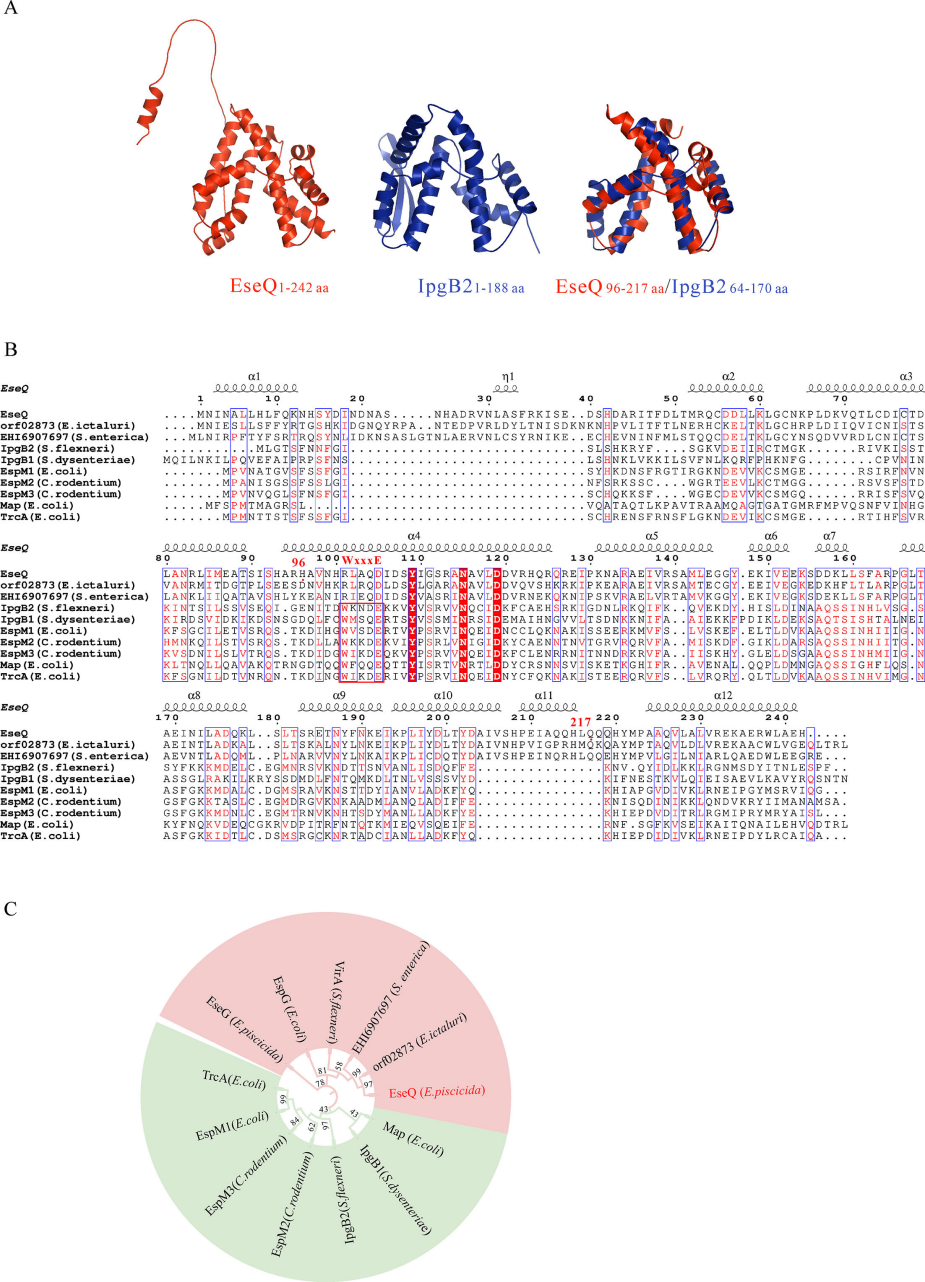
Phylogenetically, EseQ is closer to VirA than to the WxxxE effectors. (**A**) EseQ is structurally similar to IpgB2. Sequence data for EseQ in *E. piscicida* and IpgB2 in *S. flexneri* were entered into the Swiss 3D structural motif model. The amino acids 96–217 of EseQ exhibit structural similarities to the *Shigella* T3SS effector IpgB2 over the amino acids 64–170. (**B**) EseQ is not a member of the WxxxE effector family. Multiple sequence alignment and hierarchical clustering were performed on Orf2873 in *E. ictaluri*, EHI6907697 in *S. enterica,* IpgB1 in *S. dysenteriae*, IpgB2 in *S. flexneri*, EspM1, Map, and TrcA in EPEC and EspM2 and EspM3 in *C. rodentium*. The conserved motif WxxxE is shown in a red box. Similar and identical residues are highlighted in light and dark gray, respectively. (**C**) Phylogenetically, EseQ is more closely related to microtubule-destabilizing proteins, such as VirA, EspG, and EseG, than to the WxxxE family of effectors. Multiple sequences were aligned using hierarchical clustering, and a radial phylogenetic tree was constructed for VirA in *Shigella*, EspG in EPEC, and EseG and EseQ in *E. piscicida*, as well as the proteins shown in panel B.

Using the SWISS-MODEL Interactive Workspace, it was found that EseQ (amino acids [aa] 96–217) shares 27% identity with the *Shigella* T3SS effector IpgB2 (aa 64–170) in terms of their tertiary structures (Fig. 1A). IpgB2 in *Shigella flexneri*, IpgB1 in *Shigella dysenteriae*, Map, EspM1, and TrcA in enteropathogenic *Escherichia coli* (EPEC), and EspM2 and EspM3 in *Citrobacter rodentium* all share the WxxxE motif (Trp-xxx-Glu) and are potent modulators of the host’s actin cytoskeleton (highlighted by a red box in Fig. 1B) (31–34). However, multiple sequence alignment revealed that neither EseQ nor its closest homologs from *Edwardsiella ictaluri* (GenBank accession number WP_081165511) or *Salmonella enterica* (GenBank accession number EAQ8020264) possess the WxxxE motif. The homologous fragments that EseQ shares are primarily confined to amino acid residues 96–217. Identical or conserved amino acids are highlighted in red in the sequence alignment (Fig. 1B). T3SS effectors, such as EseG in *E. piscicida*, VirA in *Shigella,* and EspG in EPEC, all disrupt microtubules. The latter two were also reported to stimulate the formation of stress fibers in host cells by modulating the actin cytoskeleton (8, 29, 35). Interestingly, by the neighbor-joining phylogenetic tree, it was revealed that EseQ and its two closest homologs share a closer relationship with EspG, VirA, and EseG, which destabilize microtubules, than with the WxxxE effectors, which interfere with the actin cytoskeleton (Fig. 1C).

### EseQ binds to both tubulin and GEF-H1, destabilizing the microtubule network

**Fig 2:**
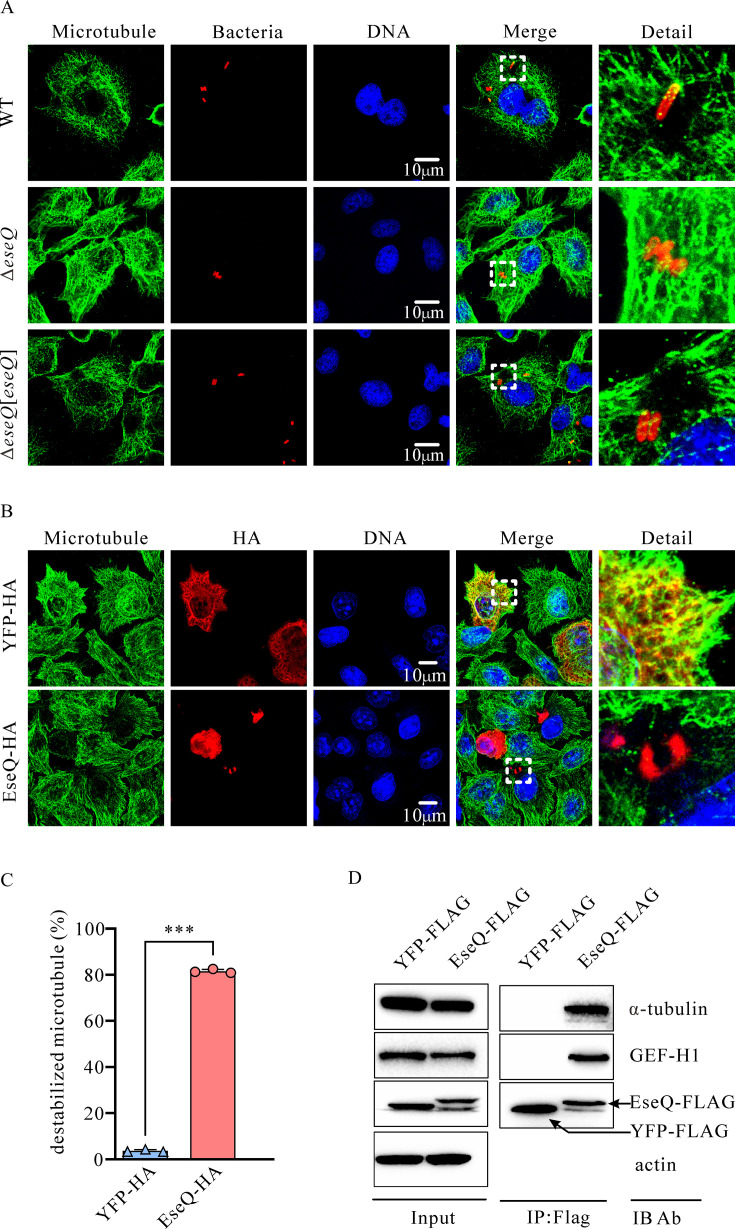
EseQ interacts with both GEF-H1 and α-tubulin and destabilizes the microtubules. (**A**) EseQ protein, delivered by *E. piscicida*, promotes microtubule destabilization. ZF4 monolayers infected with *E. piscicida* strains were stained using an immunofluorescent technique for microtubules (green, anti-α-tubulin antibody), bacteria (red, anti-LPS antibody), and nuclei (blue, DAPI). Scale bar: 10 µm. (**B**) Excessive expression of EseQ destabilizes microtubules. HeLa cells were transfected with either pCDN3.1-*eseQ*-HA or pCDN3.1-*yfp*-HA (the control). The cells were stained using an immunofluorescent technique for microtubules (green, anti-α-tubulin antibody), EseQ-HA or YFP-HA (red, anti-HA antibody), and nuclear DNA (blue, DAPI). Bar: 10 µm. (**C**) The percentage of cells with EseQ- or YFP-induced microtubule destabilization shown in panel B. This experiment was independently repeated at least three times. The means ± SD from a representative experiment are shown. A Student’s *t*-test was used to calculate the *P*-value. ***, *P* < 0.001. (**D**) EseQ binds to both tubulin and GEF-H1. HeLa cells were transfected with p-*eseQ*-3×FLAG or p-*yfp*-3×FLAG (the control), after which the cells were solubilized to prepare the cell lysates. Actin was used as a loading control for the input (left panel). These cell lysates were then immunoprecipitated using an anti-FLAG antibody. The anti-FLAG antibody precipitated both α-tubulin and GEF-H1 in the cell lysates containing EseQ-FLAG, but neither was precipitated from the cell lysates containing YFP-FLAG.

Since EseQ clusters with EspG, VirA, and EseG to form a distinct branch in the neighbor-joining phylogenetic tree, we hypothesized that EseQ might also function as a microtubule-associated effector. The zebrafish embryonic fibroblast (ZF4) cell is larger than the Epithelioma papulosum cyprini (EPC) cell and is of zebrafish origin. This makes stress fiber formation easier to observe. ZF4 monolayers were infected with *E. piscicida* strains and stained with anti-α-tubulin (green), anti-LPS (red), and DAPI (nuclear, blue). It was observed that infection with either the *E. piscicida* wild-type (WT) strain or the genetically restored knockout strain Δ*eseQ*[*eseQ*] stimulated microtubule destabilization. However, infection with the Δ*eseQ* strain failed to affect the microtubule distribution pattern (Fig. 2A). Due to the low transfection efficiency of the ZF4 cell line, HeLa cells were used to ectopically express EseQ-HA or YFP-HA, followed by immunofluorescence staining. Microtubule destabilization was observed in the vicinity of EseQ puncta. By contrast, the ectopic expression of YFP-HA had no effect on the distribution pattern of microtubules (Fig. 2B). Microtubule destabilization was observed in 81.56% ± 0.68% of HeLa cells that ectopically expressed EseQ-HA. This is dramatically and significantly higher than the 3.65% ± 0.47% observed in cells expressing YFP-HA (Fig. 2C).

GEF-H1 is the only known microtubule-associated nucleotide exchange factor (GEF) and plays a crucial role in mediating communication between microtubules and the actin cytoskeleton (21, 36). An immunoprecipitation (IP) assay was performed using HeLa cells that ectopically express either EseQ-3×FLAG or YFP-3×FLAG. Anti-FLAG-M2 affinity gel immunoprecipitation revealed that EseQ-3×FLAG specifically binds with GEF-H1. Interestingly, its interaction with α-tubulin was also detected. YFP-3×FLAG was used as a negative control, and it precipitated neither GEF-H1 nor α-tubulin (Fig. 2D, right panel). These data indicate that EseQ binds to microtubules and GEF-H1 and also destabilizes microtubule networks. These activities may release and activate GEF-H1, thereby influencing actin networks.

### EseQ triggers the formation of stress fibers

**Fig 3:**
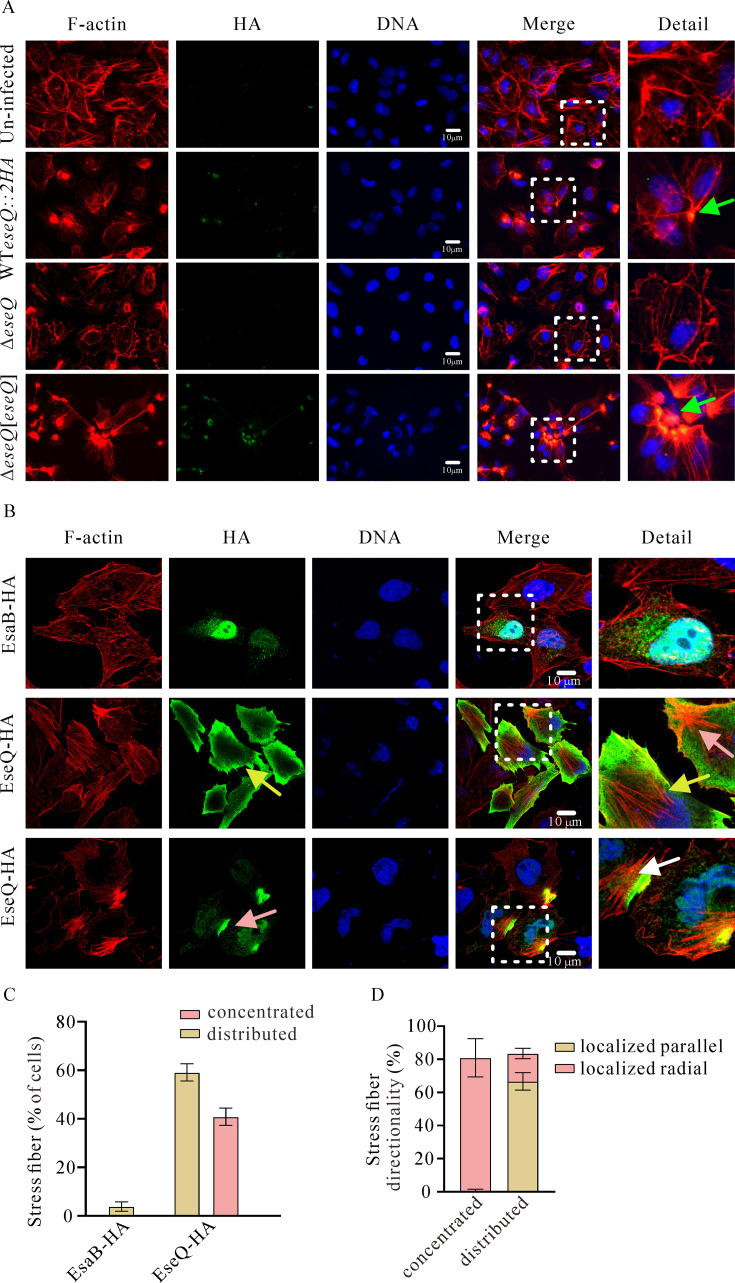
EseQ promotes the formation of stress fibers. (**A**) Translocated EseQ promotes the formation of stress fibers. ZF4 cells were infected with *E. piscicida* WT *eseQ*::2HA, Δ*eseQ*, and Δ*eseQ*[*eseQ*] strains for 3 h before being fixed and stained with TRITC-phalloidin for confocal microscopy. Scale bar: 10 µm. (**B**) Excessive expression of EseQ in HeLa cells induces stress fiber formation. HeLa cells were transfected with either pCDN3.1-*eseQ-*HA or pCDN3.1-*esaB*-HA (the control). Immunofluorescence staining was then used to stain the following in the transfected HeLa cells: F-actin (red, Alexa Fluor 555); HA (green, Alexa Fluor 488); and the nucleus (blue, DAPI). Both concentrated (pink arrow) and distributed (yellow arrow) EseQ signals were observed. Stimulated stress fibers were classified as either localized parallel (yellow arrow) or localized radial (pink arrow). Bar: 10 µm. (**C**) Quantification of stress fibers induced by concentrated and distributed EseQ, as shown in panel B. The experiment was repeated three times independently. One hundred cells were counted in triplicate. The results are expressed as the mean ± SEM. A one-way ANOVA test with multiple comparisons was used. ***, *P* < 0.001; NS, not significant. (**D**) Quantification of localized parallel and localized radial stress fibers induced by concentrated and distributed patterns of EseQ, as shown in panel B. The experiment was repeated three times independently. The results are shown as the mean ± SEM.

GEF-H1 is inactive when bound to microtubules. However, upon release, it becomes active, converting GDP-RhoA to GTP-RhoA and linking microtubule dynamics to the actin cytoskeleton (21, 36). EseQ shares a structural similarity with IpgB2, which triggers the formation of stress fibers. To investigate whether EseQ delivered by *E. piscicida* influences the host’s actin cytoskeleton, ZF4 zebrafish cell monolayers were infected with *E. piscicida* strains at an MOI of 20 for 2 h prior to immunofluorescence staining. Stress fiber formation (F-actin, red signal) was observed around the concentrated pattern of the EseQ-2HA signal (HA, green signal) in cells infected with the WT *eseQ*-2HA::*kan* strain, but not in cells infected with the Δ*eseQ* strain or uninfected ZF4 cells. The Δ*eseQ* strain’s failure to stimulate stress fiber formation was partially resolved when a wild-type copy of EseQ-2HA was expressed in the Δ*eseQ* strain (Fig. 3A).

To corroborate, EseQ-HA was ectopically expressed in HeLa cells via the introduced pCDNA-*eseQ*-HA. Stress fiber formation was observed in cells expressing EseQ-HA. EsaB, a T3SS gatekeeper protein in *E. piscicida* (15), was used as a negative control, whose expression failed to affect the distribution of F-actin (Fig. 3B). Notably, two types of EseQ signal were observed in HeLa cells: a concentrated pattern (indicated by the pink arrow) and a distributed pattern (indicated by the yellow arrow). To determine if these localization patterns had functional consequences, we quantified stress fiber formation. The results demonstrate that EseQ-HA, regardless of its concentrated or distributed pattern, robustly stimulates stress fiber formation. These patterns induced stress fiber formation with frequencies of 40.84% ± 2.91% and 59.16% ± 2.91%, respectively (Fig. 3C). Two types of stress fibers were induced: one being localized radial stress fibers (pink arrow), and the other being localized parallel stress fibers (yellow arrow). The concentrated pattern of the EseQ signal stimulated 80.35% ± 9.46% and 0.56% ± 0.80% of the localized radial and parallel stress fibers, respectively. The distributed signal pattern of EseQ stimulated 17.17% ± 2.56% and 66.64% ± 4.32% of the localized radial and parallel stress fibers, respectively (Fig. 3D). Consistently, the EseQ homolog in *E. ictaluri* (Orf2873) induced stress fiber formation in 84.90% ± 2.44% of transfected HeLa cells (image not shown). These results suggest that EseQ can stimulate stress fiber formation despite lacking the WxxxE motif.

### EseQ activates the RhoA-ROCK-MLCII pathway

**Fig 4:**
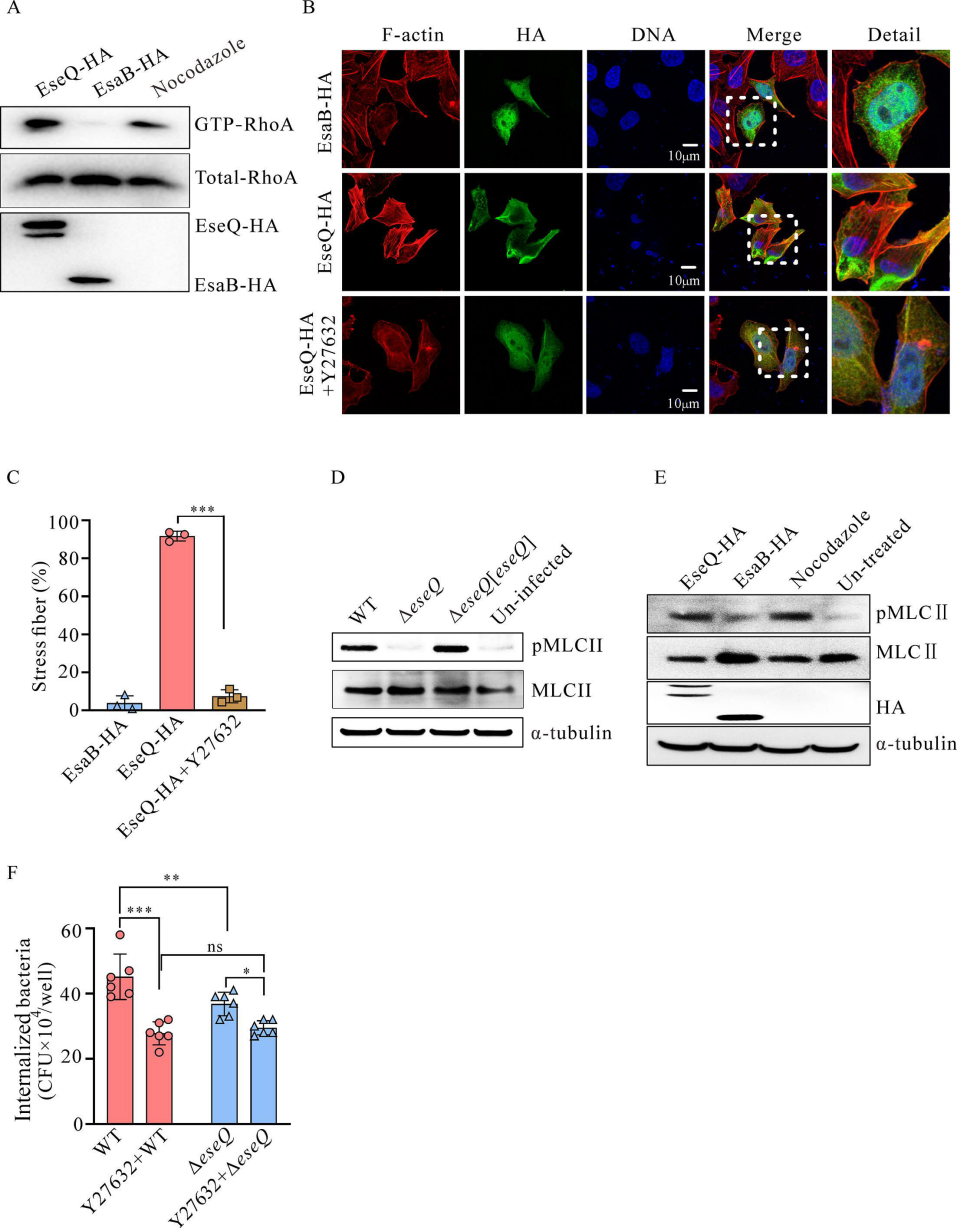
EseQ activates the RhoA-ROCK-MLCII signaling pathway. (**A**) EseQ promotes the conversion of GDP-bound RhoA to GTP-bound RhoA. GTP-bound RhoA was precipitated in a Rhotekin pull-down assay using HeLa cells that had been transfected with either pCDNA3.1-*eseQ*-HA or pCDNA3.1-*esaB*-HA. HeLa cells pretreated with nocodazole were used as a positive control. The precipitates (GTP-RhoA) and total cell lysates (total-RhoA) were probed with an anti-RhoA antibody. (**B**) Stress fibers induced by EseQ can be inhibited by ROCK inhibitors. HeLa cells were transfected with either pCDN3.1-*eseQ*-HA or pCDN3.1-*esaB*-HA (the control). Prior to transfection, the HeLa cell monolayers were treated with 10 µM of the ROCK inhibitor Y27632 for 1 h. Afterward, the cells were stained for F-actin (red, Alexa Fluor 555), HA (green, Alexa Fluor 488), and nuclei (blue, DAPI). (**C**) Quantification of stress fiber-positive cells, as shown in panel B. A one-way ANOVA with a multiple comparisons test was used. The experiment was repeated three times independently, and the data shown here are representative, with the mean ± SD indicated. ***, *P* < 0.001. (**D**) Delivery of EseQ by *E. piscicida* increases the levels of phosphorylated MLCII protein in ZF4 cells. Cell lysates from infected ZF4 monolayers were probed with anti-MLCII and anti-pMLCII antibodies; tubulin was used as a loading control. This experiment was repeated at least three times independently, and the results shown here are representative. (**E**) EseQ alone induces increased levels of phosphorylated MLCII protein in HeLa cells. The cells were either transfected with pCDN3.1-*eseQ*-HA or pCDN3.1-*esaB*-HA (the control), or pretreated with 10 µM nocodazole for 1 h (the positive control). Cell lysates were probed with anti-MLCII and anti-pMLCII antibodies, with tubulin used as a loading control. This experiment was independently repeated at least three times, and the data shown here are representative. (**F**) EseQ promotes the invasion of *E. piscicida* via the RhoA-ROCK pathway. Prior to infection with either the WT or the Δ*eseQ* strain of *E. piscicida*, EPC monolayers were pretreated with or without the ROCK inhibitor Y27632. One hour after infection, the monolayers were washed and lysed in order to count the number of bacteria associated with the cells via plating. The means ± SD of a representative experiment are shown. A one-way ANOVA test with multiple comparisons was used. ns, not significant; *, *P* < 0.05; **, *P* < 0.01; ***, *P* < 0.001.

The Rho family of small GTPases is a key regulator of actin dynamics. These include RhoA, which plays a crucial role in forming contractile actomyosin stress fibers (29). To determine whether the activation of RhoA by EseQ triggers the formation of stress fibers, we ectopically expressed EseQ-HA and EsaB-HA in HeLa cells. Cells that had been pretreated with 20 µM nocodazole were used as a positive control. Nocodazole induces the release of GEF-H1 through microtubule depolymerization. The resulting free GEF-H1 then activates RhoA, thereby stimulating the formation of stress fibers (28). The level of GTP-RhoA protein (the active form of RhoA) in HeLa cells was determined using a Rhotekin-RBD bead pull-down assay, as described by Hiyoshi et al. (27). Both nocodazole pretreatment and EseQ expression resulted in substantial levels of RhoA-GTP, while comparatively little RhoA-GTP was observed in cells that expressed EsaB (Fig. 4A). These results demonstrate that RhoA is activated by the transient expression of EseQ.

ROCK (Rho-associated kinase) is one of the key downstream effectors of RhoA that mediates stress fiber formation (17). Y27632 is a ROCK inhibitor (27). HeLa cells were pretreated with Y27632 for 1 h prior to transfection with pCDNA-EseQ-HA. Immunofluorescence analysis revealed that Y27632 effectively inhibited stress fiber formation stimulated by EseQ (Fig. 4B). The ectopic expression of EseQ or EsaB in HeLa cells stimulated the formation of stress fibers in 91.74% ± 3.38% and 3.78% ± 2.87% of cells, respectively. Pretreatment with Y27632 before transfection with pCDNA-EseQ-HA reduced stress fiber formation to 7.44% of cells, which corresponds to an 84.29% ± 4.81% decrease compared to untreated controls. This attenuation was comparable to that observed in the EsaB control group (Fig. 4C). These results demonstrate that EseQ stimulates stress fiber formation via activation of the RhoA-ROCK pathway.

Signaling downstream of the small GTPase Rho regulates cytoskeletal contractility through the ROCK-mediated accumulation of phosphorylated MLCII (37). To ascertain whether translocated EseQ increases the steady-state levels of phosphorylated MLCII, immunoblotting analysis was performed on ZF4 monolayers infected with *E. piscicida* strains. Similar levels of total MLCII were detected in the cell lysate of each infection; however, the levels of phosphorylated MLCII protein decreased markedly when cells were infected with the Δ*eseQ* strain compared to the WT *E. piscicida* strain or the Δ*eseQ*[*eseQ*] strain (Fig. 4D). To corroborate this, pMLCII levels in HeLa cells that ectopically express either EseB-HA or EseQ-HA were examined. HeLa cells pretreated with nocodazole were used as the positive control, while untreated cells served as the negative control. It was found that overexpressing EseQ alone is sufficient to induce MLCII phosphorylation (Fig. 4E). Together, these data confirm that EseQ alone triggers the formation of actin stress fibers by regulating the RhoA-ROCK-pMLCII signaling pathway.

The SPI-1 effectors SipA and SipC manipulate the host cell’s actin cytoskeleton to facilitate *Salmonella* invasion (19). EPC cells are most often used in *Edwardsiella piscicida* adhesion assays (3, 9, 38). To determine if actin polymerization mediated by EseQ contributes to *E. piscicida* invasion, EPC monolayers were infected with the WT strain and the Δ*eseQ* strain at an MOI of 10, and the number of cell-associated bacteria was determined at 1 h post-injection (hpi). Compared to the WT strain, depletion of EseQ was found to significantly reduce invasion. Meanwhile, no significant difference in invasion was detected when the monolayers were pretreated with the ROCK inhibitor Y27632 (Fig. 4G). Therefore, EseQ facilitates the invasion of *E. piscicida* into EPC cells by manipulating the actin cytoskeleton through the RhoA-ROCK pathway.

### EseQ disrupts the epithelial barrier

**Fig 5:**
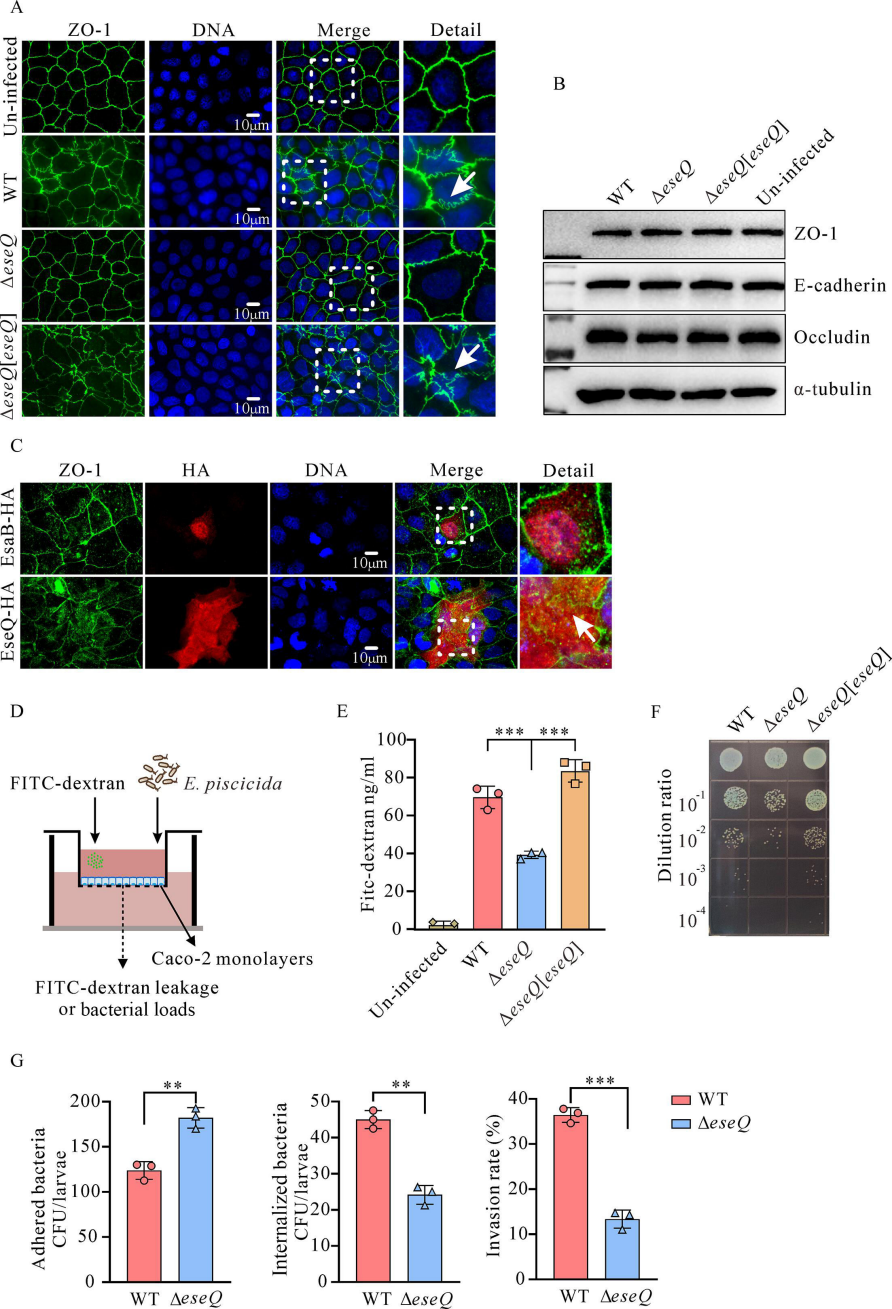
Disruption of the tight junctions of the apical Caco-2 cells by the translocation of EseQ increases the penetration of *E. piscicida*. (**A**) EseQ delivered by *E. piscicida* deforms the morphology of tight junctions mediated by ZO-1 in Caco-2 cell monolayers. Monolayers of Caco-2 cells infected with the WT *E. piscicida*, the Δ*eseQ* strain, and the Δ*eseQ*[*eseQ*] strain were fixed and stained with an immunofluorescent dye to visualize ZO-1. Bar: 10 µm. (**B**) Infection with *E. piscicida* does not remove tight junction-associated proteins. Immunoblotting was performed on ZO-1, E-cadherin, and occludin in Caco-2 cells infected with *E. piscicida* strains. α-Tubulin was used as a loading control to ensure that similar quantities of protein were loaded into each lane. (**C**) EseQ alone induces alterations in tight junctions. Caco-2 cells that had been transfected with either pCDNA-*eseQ*-HA or pCDNA-*esaB*-HA were subjected to immunostaining using an anti-ZO-1 antibody (green), an anti-HA antibody (red), and DAPI to stain the nuclei (blue). Scale bar: 10 µm. (**D**) Schematic diagram showing *E. piscicida* infection in an *in vitro* epithelial barrier model. Caco-2 cells were evenly seeded into Transwell inserts, and fresh culture medium was added to the outer chamber. The medium was replaced every other day. Approximately 15 days after seeding, when a difference in liquid level was observed between the inner and outer chambers of the Transwell, the infection was initiated. (**E**) EseQ protein, which is delivered by *E. piscicida*, alters the permeability of the polarized epithelial barrier. Caco-2 monolayers were infected with *E. piscicida* strains for 12 h prior to the application of FITC-dextran in order to examine permeability. The means ± SD of a representative experiment are shown. A one-way ANOVA test with multiple comparisons was used. ***, *P* < 0.001. (**F**) EseQ facilitates the penetration of Caco-2 monolayers. The monolayers were infected with *E. piscicida* strains for 12 h, after which the liquid from the lower chamber of the Transwell was serially diluted and spotted onto TSA plates containing colistin in order to quantify the penetration of *E. piscicida*. (**G**) EseQ facilitates the invasion of zebrafish larvae by the bacterium *E. piscicida*. Four-day-old zebrafish larvae were infected with *E. piscicida* strains at a concentration of 2 × 10^6^ CFU/mL by immersion in a bath. The larvae were ground up, and the associated bacteria were quantified by CFU enumeration. The ratio of internalized bacteria (1 hpi) to adherent bacteria (0 hpi) was then calculated. The means ± SD from a representative experiment are presented. A Student’s *t*-test was used to calculate the *P*-value. **, *P* < 0.01; ***, *P* < 0.001.

Tight junctions (TJs) are primarily composed of transmembrane proteins known as claudins and occludins (39). Disruption to tight junctions can result in a loss of intestinal epithelial barrier homeostasis (39–41). The adaptor protein zonula occludens-1 (ZO-1) facilitates the close interaction between tight junctions and the actin cytoskeleton (39, 40). Caco-2 cells, which can form polarized monolayers with tight junctions, were used to evaluate the epithelial barrier and bacterial translocation. The ZO-1-mediated tight junctions between Caco-2 cells were stained immunofluorescently after being infected with *E. piscicida* strains at an MOI of 20 for 8 h. It was found that infection with either the WT strain or the Δ*eseQ*[*eseQ*] strain resulted in greater deformability of the ZO-1 signaling pattern. This manifested as greater deformation of the ZO-1 geometry, which resulted in multiple depressions and protrusions forming. In contrast, the Δ*eseQ* strain only caused minor alterations to the ZO-1-mediated tight junction (Fig. 5A). However, immunoblotting revealed similar steady-state levels of ZO-1, E-cadherin, and occludin in Caco-2 cells infected with the WT strain, the Δ*eseQ* strain, the Δ*eseQ*[*eseQ*] strain, and the uninfected control (Fig. 5B). To corroborate that the formation of multiple depressions and protrusions depends on EseQ alone, EseQ-HA or EsaB-HA was ectopically expressed in Caco-2 cells, after which the integrity of the tight junctions was examined using immunofluorescence staining with an anti-ZO-1 antibody. It was revealed that EseQ alone is sufficient to alter the tight junctions, resulting in greater deformability of the ZO-1 signaling pattern (Fig. 5C). These results show that although translocated EseQ stimulates barrier deformation, this deformation is not caused by the degradation of tight junction proteins.

The effect of EseQ on the epithelial barrier was further investigated using an *in vitro* epithelial barrier Transwell model (Fig. 5D). The integrity of the epithelial barrier was evaluated by measuring the leakage of FITC-dextran in Caco-2 cells infected with *E. piscicida* strains at an MOI of 20 for 12 h. A solution of FITC-dextran (4.0 kDa) at a concentration of 0.1 mg/mL was applied to the apical side of polarized Caco-2 cells. One hour later, FITC-dextran leakage was monitored. Upon infection with the WT strain, the amount of basolateral dextran increased dramatically (69.61 ± 4.79 ng/mL), compared to the Δ*eseQ* strain (39.28 ± 1.56 ng/mL) and uninfected controls (2.13 ± 1.69 ng/mL). Adding a functional copy of EseQ to the Δ*eseQ* strain restored basolateral dextran leakage to a level similar to that observed in cells infected with the WT strain (83.61 ± 4.87 ng/mL) (Fig. 5E). These data suggest that EseQ disrupts ZO-1-mediated tight junctions, thereby causing epithelial barrier dysfunction. At the same time, bacterial penetration was evaluated by spot plating serial dilutions of the liquid from the lower chamber of the Transwell system. Compared to the Δ*eseQ* strain, both the wild-type and complemented strains exhibited increased bacterial translocation, suggesting that EseQ enhances the ability of *E. piscicida* to penetrate the epithelial barrier (Fig. 5F).

### The EseQ protein enables *Edwardsiella* to invade fish larvae

In order to examine whether EseQ facilitates the invasion of *E. piscicida*, we conducted an *in vivo* infection assay on zebrafish larvae. Four-day-old zebrafish larvae were infected with *E. piscicida* by being immersed in a solution containing the bacterium. At time 0, the adhesion rates were 124 ± 8 CFU/larva for the WT strain and 182 ± 9 CFU/larva for the Δ*eseQ* strain (Fig. 5F, left panel). One hour post-injection, the invasion was 45 ± 2 CFU/larva for the wild-type strain and 24 ± 2 CFU/larva for the Δ*eseQ* strain (Fig. 5F, middle panel). The invasion rates (internalized versus adhered) for the wild-type and Δ*eseQ* strains were 36.4%2 ± 1.33% and 13.33% ± 1.64%, respectively (Fig. 5F, right panel). These results demonstrate that EseQ inhibits adhesion while promoting the invasion of *E. piscicida* in zebrafish.

## DISCUSSION

The cell cytoskeleton and its associated proteins are key targets for bacterial effectors that manipulate actin or microtubules in order to facilitate invasion and spread (24, 42). This study demonstrated that EseQ destabilizes microtubules, thereby releasing GEF-H1 and activating the RhoA signaling pathway. This triggers the formation of stress fibers via the RhoA-ROCK-MLCII pathway, resulting in epithelial barrier dysfunction and increased invasion.

EseQ can induce the formation of stress fibers in host cells. This function is similar to that of the T3SS effectors IpgB2, IpgB1, TrcA, EspM1, EspM2, and EspM3 and Map. Unlike *E. piscicida* EseQ, each of these contains a conserved WxxxE motif (32, 34, 43, 44). In *V. parahaemolyticus*, the T3SS effector VopO binds directly to GEF-H1, thereby activating the RhoA-ROCK pathway and inducing stress fibers (28). This process is very similar to that of EseQ, which also binds to GEF-H1. Additionally, the *Bartonella* type IV secretion effector BepC induces stress fiber formation by activating GEF-H1 (45). However, like *E. piscicida* EseQ, none of these three effectors possess a WxxxE motif. IpgB2 activates Rho-dependent signaling pathways by mimicking GEFs (34). However, EseQ triggers the destabilization of microtubules, resulting in the release of GEF-H1. This then converts GDP-RhoA to GTP-RhoA, thereby stimulating the formation of stress fibers via subsequent RhoA and ROCK effectors. Consistent results from phylogenetic analysis revealed that EseQ is more closely related to EspG in EPEC, VirA in *Shigella*, and EseG in *Edwardsiella*. All of these proteins stimulate microtubule destabilization. This explains why EseQ promotes microtubule depolymerization.

A common strategy employed by intracellular bacterial pathogens is to manipulate the host cell’s actin cytoskeleton to facilitate invasion (17, 19). For example, the proteins *Shigella* IpaC, IpgB1, and IpgD promote the polymerization of actin, which drives the extension of the cell membrane surrounding invading bacteria and facilitates their entry into epithelial cells (46, 47). The invasion rate of the Δ*eseQ* strain is lower than that of the wild-type *E. piscicida* strain. *E. piscicida* EseQ activates the RhoA-ROCK-pMLCII signaling pathway and induces stress fiber formation, thereby promoting invasion. The higher invasion rates observed in zebrafish larvae may be due to the apical cell barrier becoming dysfunctional through rearrangement of the actin cytoskeleton upon EseQ translocation, which promotes *E. piscicida* invasion into the submucosa.

The actin cytoskeleton plays a critical role in maintaining epithelial barrier function (46). Bacterial effectors or toxins target Rho GTPases, disrupting cell-to-cell junctions and promoting bacterial translocation (24, 40, 48, 49). *Salmonella* T3SS effectors, such as SopB, SopE, SopE2, and SipA, disrupt the tight junctions between neighboring apical cells (48). *Shigella* disrupts the integrity of the intestinal epithelial barrier by secreting the serine protease SepA, which activates the actin-depolymerizing factor cofilin (50). Map in EPEC disrupts the integrity of junctions by preventing TJ proteins from being recruited to newly formed junctions (44). The Caco-2 cell model of polarized epithelial cells shows that EseQ causes barrier dysfunction by altering the structure of the tight junctions mediated by the ZO-1 protein, rather than by degrading ZO-1, E-cadherin, or occludin. This deformation, induced by EseQ, may be due to an altered actin cytoskeleton. This is because ZO-1 acts as an adaptor linking the actin cytoskeleton to cellular tight junctions (39, 40).

Currently, we have no idea whether EseQ is translocated when *E. piscicida* adheres to host epithelial cells. *E. piscicida* has only one T3SS gene cluster, which contains almost all the genes homologous to those of SPI-2 (*Salmonella* pathogenicity island 2) (3). SPI-1 translocates T3SS1 effectors to promote invasion (19). We speculate that EseQ delivered by *E. piscicida* inside the vacuole induces stress fibers, leading to the formation of membrane ruffles. This facilitates the internalization of a second batch of *E. piscicida*. Further study is required to determine whether EseQ is translocated upon attachment and plays a role in the very early stages of invasion.

An intriguing finding is that EseQ enhances invasion yet reduces adhesion. Many bacterial pathogens primarily invade non-phagocytic cells via the “adhesin-integrin-cytoskeleton” pathway. For instance, *Staphylococcus aureus* binds to integrin α5β1, thereby promoting adhesion (51). Similarly, *Listeria monocytogenes* utilizes internalin A to bind to the host E-cadherin receptor, thereby achieving adhesion and invasion (52). These receptors on host cells require the maintenance of cytoskeletal homeostasis and the integrity of the intestinal barrier. However, EseQ disrupts both of these processes. It has been speculated that the reduction in adhesion may result from EseQ interfering with host cell surface receptors or adhesins that facilitate attachment, thereby shifting the balance toward a more rapid and dedicated invasion pathway.

**Fig 6:**
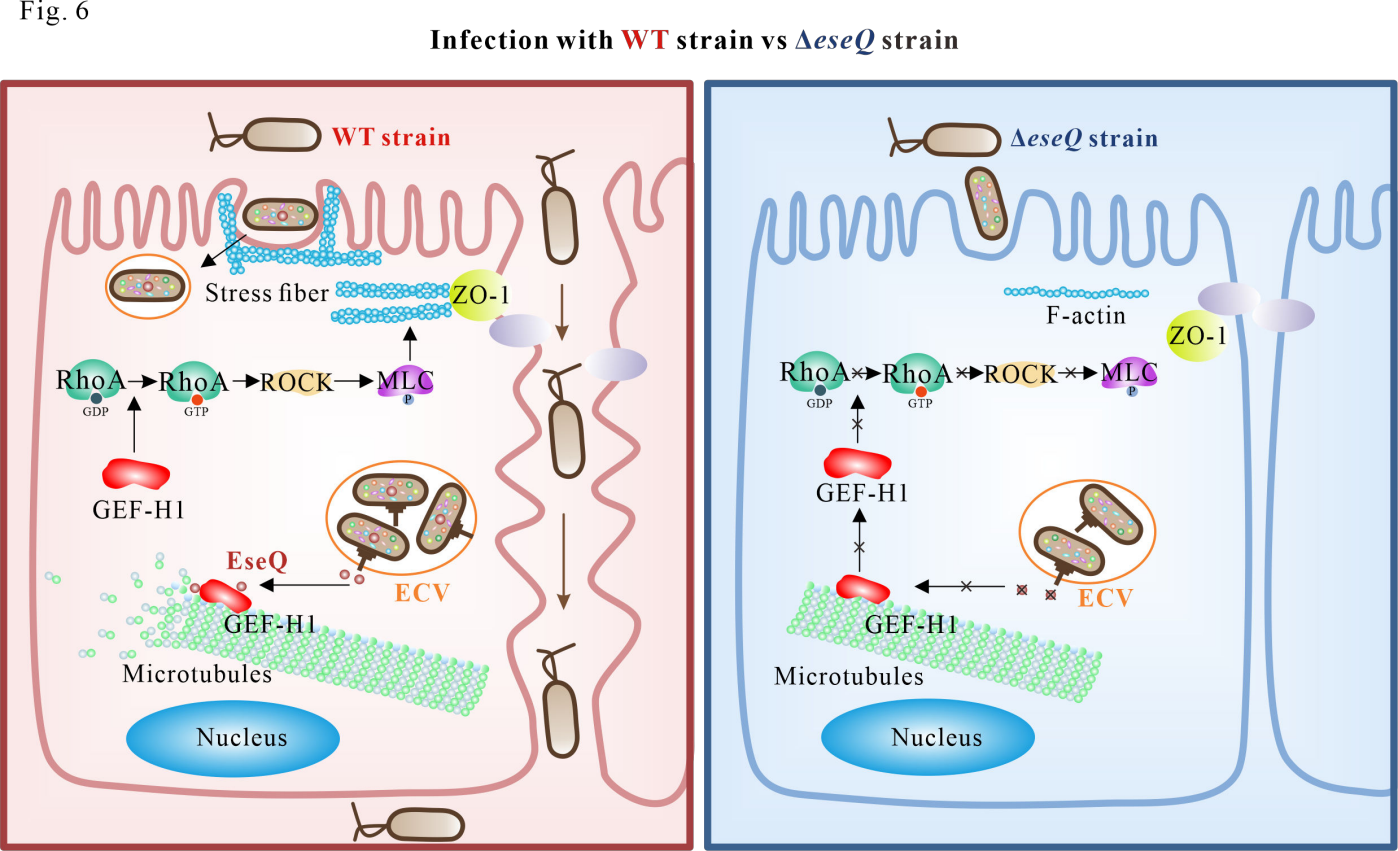
The schematic diagram shows the link between microtubule destabilization induced by EseQ, stress fiber formation, and epithelial barrier dysfunction. *E. piscicida* delivers EseQ from the *Edwardsiella*-containing vacuole (ECV) into the host cell, where it interacts with GEF-H1 and microtubules. This process destabilizes the microtubule structure and releases GEF-H1. Free GEF-H1 then activates RhoA by converting it from its inactive GDP-bound form to its active GTP-bound form. This activates its downstream effector, ROCK, resulting in the phosphorylation of MLCII and stress fiber formation. This process facilitates the internalization of *E. piscicida* into host epithelial cells. Meanwhile, EseQ alters the structure of ZO-1-mediated TJs, which disrupt the epithelial barrier and promote invasion. Overall, EseQ destabilizes microtubules, activates the RhoA-ROCK pathway, and exerts a regulatory effect on F-actin. This disrupts the structure of ZO-1-mediated TJs and facilitates invasion.

In *Salmonella*, SipC and SipA are sequentially translocated via the T3SS. SipC nucleates actin filaments, which are stabilized by SipA binding. This process is crucial for forming the cytoskeletal structure that enables invasion and survival (19). It is tempting to speculate that other T3SS effectors in *E. piscicida* work alongside EseQ to manipulate the cell’s cytoskeleton, thereby facilitating invasion. Figure 6 illustrates the functional dissection of EseQ. Specifically, EseQ interacts with both GEF-H1 and microtubules. By destabilizing the microtubules, GEF-H1 is then released and becomes activated. Active GEF-H1 then activates the RhoA-ROCK-pMLCII signaling pathway, resulting in the formation of stress fibers. Meanwhile, EseQ alters the structure of TJs, which are linked to the actin cytoskeleton via a protein called ZO-1, thereby disrupting the epithelial barrier. Dysfunction of the epithelial barrier increases the likelihood of *E. piscicida* invasion. Our study is the first to establish a link between EseQ’s role in altering the cell cytoskeleton, causing epithelial barrier dysfunction, and facilitating bacterial invasion. This improves our understanding of the pathogenic mechanism of *E. piscicida*.

## MATERIALS AND METHODS

### Bacterial strains, cell lines, and cultivation

The bacterial strains and plasmids used in this study are listed in Table 1. *Edwardsiella piscicida* PPD130/91 and its derivative strains were grown statically in tryptic soy broth (BD Biosciences) at 28°C. When necessary, the medium was supplemented with the appropriate antibiotics at the following concentrations: 12.5 µg/mL colistin (Col; Sigma), 15 µg/mL tetracycline (Tet; Amresco), and 50 µg/mL gentamicin (Gm; Amresco).

**TABLE 1:** Strains and plasmids used in this study^*a*^

Strain or plasmid	Description and/or genotype	Reference or source
*E. piscicida* strains		
PPD130/91	Wild-type strain, Col^r^	29
∆*eseQ*	PPD130/91, *eseQ* in-frame deletion of aa 1–242	12
∆*eseQ*[*eseQ*]	Δ*eseQ* transformed with pACYC184-*eseQ*-HA, Tet^r^	12
WT *eseQ*::2HA	PPD130/91, *eseQ* gene chromosomally tagged with 2HA at its C terminus	12
Plasmids		
pCDNA3.1	CMV promoter, Amp^r^ Neo^r^	Invitrogen
p-3×FLAG	CMV promoter, Amp^r^ Neo^r^	Invitrogen
pCDNA-*eseQ*-HA	pCDNA3.1 with HA tag at the C terminus of *eseQ*	12
pCDNA-*esaB*-HA	pCDNA3.1 with HA tag at the C terminus of *esaB*	This study
p-*eseQ*-3×FLAG	p-3×FLAG inserted with 3×FLAG tag at the C terminus of *eseQ*	This study
p-*yfp*-3×FLAG	p-3×FLAG inserted with 3×FLAG tag at the C terminus of *yfp*	53

^*a*^ Amp, ampicillin; r, resistance.

HeLa cells were grown at 37°C in Dulbecco’s modified Eagle’s medium (Gibco) with 10% fetal bovine serum (FBS). Zebrafish embryonic fibroblast (ZF4) cells were cultured at 28°C in Dulbecco’s Modified Eagle’s minimum-Ham’s F12 medium (Gibco) with 10% FBS. EPC cells were grown at 28°C in M199 medium (M199, Gibco) with 10% FBS. Caco-2 cells were grown at 37°C in Minimum Essential Medium (Gibco) with 20% FBS. The Caco-2 cells were grown at 35°C for infection with *E. piscicida* strains. All the cells were cultured under a 5% (vol/vol) CO_2_ atmosphere.

### Treatment of HeLa cells with an inhibitor

Twenty-four hours prior to transfection, HeLa cells were seeded at a density of 1.0 × 10^5^ cells per well in 24-well tissue culture plates. The cells were then transiently transfected with 2 µL of Fugene 6 (Roche) and 1.0 µg of pcDNA-*esaQ*-HA or pcDNA-*esaB*-HA (primers used in their construction are shown in Table 2), optimized according to the manufacturer’s instructions. The cells were incubated at 37°C in 5% CO_2_ for 18 h prior to fixation. To inhibit the formation of stress fibers, the cells were pretreated with 10 µM Y27632 (Sigma-Aldrich) for 1 h before fixation.

**TABLE 2:** Oligonucleotides used in this study

Primer	Sequence (5′-3′)
pCDNA-*esaB*-HA-for	ATAAGAATGCGGCCGCATGCACCCGATACGCAGC
pCDNA-*esaB*-HA-rev	CGGGATCCTCAAGCGTAATCTGGAACATCGTATGGGTATGCATACCTCCCAATGAACGGG
pCDNA-*eseQ*-HA-for	GGGGTACCGCCACCATGAATATTAACGCCCTGCTGCA
pCDNA-*eseQ*-HA-rev	CGGAATTCTCAAGCGTAATCTGGAACATCGTATGGGTAGCGGGC ATGAGAGATAGACGT
p-*eseQ*-3×FLAG-for	GGAATTCATGAATATTAACGCCCTGCTGCA
p-*eseQ*-3×FLAG-rev	CGGGATCCATGTTCCGCGAGCCACC
p-*yfp*-HA-for	CGGGATCCATGGTGAGCAAGGGCGAG
p-*yfp*-HA -rev	GGAATTCTTAAGCGTAATCTGGAACATCGTATGGGTACTTGTACAGCTCGTCCATGCC

### Immunofluorescence microscopy

The ZF4 or Caco-2 cell lines were infected with the WT *E. piscicida*, the Δ*eseQ,* and Δ*eseQ*[*eseQ*] strains at an MOI of 20 for 2 h or 8 h. The HeLa cells or Caco-2 cells were transfected with pcDNA-esaQ-HA or pcDNA-esaB-HA for 20 h. The monolayers that had been transfected or infected were washed three times with prewarmed PBS. For immunofluorescence staining of microtubules, the monolayers were then fixed in either cold methanol for 1 min or 4% paraformaldehyde in PBS (pH 7.4) for 20 min. After permeabilization with 0.2% Triton X-100 in PBS for 15 min, the monolayers were subjected to immunofluorescence staining. The following antibodies were used at the indicated dilutions: TRITC-phalloidin-Alexa Fluor 555 (1:500, Yeasen); rabbit anti-HA antibody (1:300, Cell Signaling Technology); rabbit anti-ZO-1 antibody (1:300, Cell Signaling Technology); rabbit anti-α-tubulin antibody (1:500, Abcam); mouse anti-LPS (*E. piscicida*) antibody (1:500, 53); mouse anti-HA antibody (1:300, Cell Signaling Technology); goat anti-rabbit IgG Alexa Fluor 488 (1:200, Molecular Probes); and goat anti-mouse IgG Alexa Fluor 594 (1:200, Molecular Probes). Images were captured using a confocal laser scanning microscope (NLO-LSM 710, Carl Zeiss).

### RhoA activation assay

RhoA activation was analyzed using a G-LISA RhoA Activation Assay Biochem Kit (Cytoskeleton, Inc.). In brief, the following treatments were performed on HeLa cells: transfection with either pcDNA-*eseQ*-HA or pcDNA-*esaB*-HA (the negative control) and treatment with 20 µM nocodazole (Sigma-Aldrich) for 30 min (the positive control). RhoA activation was evaluated using a pull-down assay. Levels of GTP-RhoA protein in the transfected HeLa cells were estimated using a Rhotekin pull-down assay with Rhotekin-RBD beads. The samples were then subjected to immunoblotting using a rabbit anti-RhoA antibody (dilution 1:3,000, Cytoskeleton, Inc.).

### MLCII phosphorylation assay

ZF4 cells were infected with *E. piscicida* strains at an MOI of 20 for 3 h.

HeLa cells were transfected with pcDNA-*eseQ*-HA or pcDNA-*esaB*-HA. Pretreatment with nocodazole was used as a positive control. The cell lysates were then subjected to immunoblotting using p (Thr18/Ser19)-MLCII or MLCII antibody (each diluted at a ratio of 1:3,000; Cell Signaling Technology).

### IP assay

HeLa cells grown in 100 mm diameter culture dishes were transfected with either 10 µg of p-*eseQ*-3×FLAG or 10 µg of p-*yfp*-3×FLAG (54) using 20 µL of Lipofectamine 2000 (Invitrogen) for 20 h. After this time, the cells were washed with cold PBS and scraped. The cells were washed three times with cold PBS, then lysed for 30 min in a modified IP lysis buffer (25 mM Tris-HCl, pH 7.4; 150 mM NaCl; 1 mM EDTA; 1% NP-40; and 5% glycerol) (Pierce) supplemented with a cocktail of protease inhibitors (aspartic acid, cysteine, and serine proteases) (Thermo). The resulting homogenate was then centrifuged, and the supernatant was incubated with anti-FLAG-conjugated agarose beads (Invitrogen) overnight at 4°C with gentle rotation. The immunoprecipitates were then extensively washed with ice-cold IP buffer.

### Immunoblotting assay

The proteins were separated by SDS-PAGE and transferred to polyvinylidene fluoride membranes (Merck Millipore). The membranes were then probed with the following antibodies: rabbit anti-RhoA (1:3,000, Cytoskeleton), rabbit anti-MLCII (1:3,000, CST), rabbit anti-p-MLCII (Thr18/Ser19) (1:3,000, CST), rabbit anti-ZO-1 (1:3,000, CST), mouse anti-E-cadherin (1:3,000, CST), rabbit anti-occludin (1:3,000, CST), rabbit anti-GEF-H1 (1:3,000, Abcam), rabbit anti-β-actin (1:3,000, ABclonal), rabbit anti-α-tubulin (1:3,000, Abcam), mouse anti-FLAG (1:3,000, Sigma), and rabbit anti-HA (1:3,000, CST). This was followed by the addition of horseradish peroxidase-conjugated goat anti-rabbit IgG (1:5,000, Millipore) or goat anti-mouse IgG (1:5,000, Millipore). The antigen-antibody complexes were then detected using SuperSignal West Pico chemiluminescent substrate (Thermo Fisher Scientific) and imaged using a ChemiDoc MP imaging system (Bio-Rad Laboratories).

### FITC-dextran leakage and bacterial penetration assay

The Caco-2 monolayers were infected with the *E. piscicida* WT, Δ*eseQ*, and Δ*eseQ*[*eseQ*] strains at an MOI of 20 for 20 h at 35°C. The upper chamber of the Transwell plate was filled with 1.0 mL of PBS supplemented with 0.1 mg/mL FITC-dextran 4 kDa (Sigma-Aldrich, St. Louis, MO, USA), and 1.0 mL of PBS was added to the lower chamber. The plate was then incubated at 35°C for 1 h. The concentration of FITC-dextran FD4 in the lower chamber was determined using an ELx800 microplate reader (BioTek, USA). In addition, the liquids in the lower chamber of the Transwell were collected and diluted serially. Five microliters of each was spotted onto TSA plates supplemented with colistin and incubated at 28°C overnight. This assay quantifies the efficiency with which different bacterial strains can translocate across epithelial cell monolayers.

### Invasion to EPC monolayers

Twenty-four hours prior to infection, EPC cell monolayers were seeded at a density of 5 × 10^5^ cells per well in 24-well plates. EPC cells were pretreated with 10 µM Y27632 inhibitor or a respective control reagent 1 h before infection. EPC cells were infected with the *E. piscicida* WT, Δ*eseQ,* and Δ*eseQ*[*eseQ*] strains at an MOI of 20. The plate was then centrifuged at 170 × *g* for 5 min at room temperature. The infection was then maintained at 25°C in a 5% CO_2_ incubator for 30 min (zero post-infection). The monolayers were washed three times with prewarmed PBS and lysed with 0.2% Triton X-100, and the bacteria were quantified by plating a dilution series onto TSA plates. To measure the numbers of internalized bacteria, the medium was then replaced with prewarmed culture medium supplemented with 100 µg/mL gentamicin to kill any remaining extracellular bacteria. The infection was maintained for 1 h. Subsequently, the EPC monolayers were washed three times with prewarmed PBS and lysed with 0.2% Triton X-100 for plating.

### Zebrafish larva invasion assay

On day 4 post-fertilization, wild-type AB zebrafish larvae were infected with *E. piscicida* strains by bath immersion at a concentration of 2 × 10^6^ CFU/mL. The larvae were then incubated at 28°C for 30 min. After this time, they were washed three times and ground with a clear glass pestle in 1.0 mL of PBS. The bacteria were quantified by plating the dilution series onto TSA plates. Forty larvae were collected per infection. The remainder were held for an additional hour to analyze larva-associated internalized bacteria. The experiment was repeated at least three times in triplicate.

### Statistical analysis

All experiments were independently repeated at least three times, and the results are presented as the mean ± standard error of the mean or the mean ± standard deviation. Comparisons between groups were made using either a one-way ANOVA with multiple comparisons or a Student’s *t*-test. *P*-values below 0.05 were considered significant.
